# Assessment of copy number variations in the brain genome of schizophrenia patients

**DOI:** 10.1186/s13039-015-0144-5

**Published:** 2015-07-01

**Authors:** Miwako Sakai, Yuichiro Watanabe, Toshiyuki Someya, Kazuaki Araki, Masako Shibuya, Kazuhiro Niizato, Kenichi Oshima, Yasuto Kunii, Hirooki Yabe, Junya Matsumoto, Akira Wada, Mizuki Hino, Takeshi Hashimoto, Akitoyo Hishimoto, Noboru Kitamura, Shuji Iritani, Osamu Shirakawa, Kiyoshi Maeda, Akinori Miyashita, Shin-ichi Niwa, Hitoshi Takahashi, Akiyoshi Kakita, Ryozo Kuwano, Hiroyuki Nawa

**Affiliations:** Department of Molecular Neurobiology, Brain Research Institute, Niigata University, 1-757, Asahimachi-dori, 951-8585 Niigata, Japan; Department of Psychiatry, Graduate School of Medical and Dental Sciences, Niigata University, 1-757, Asahimachi-dori, 951-8510 Niigata, Japan; Matsuzawa Hospital, Setagaya-ku, 156-0057 Tokyo, Japan; Departments of Neuropsychiatry, Fukushima Medical University School of Medicine, 960-1295 Fukushima, Japan; Division of Psychiatry and Neurology, Kobe University Graduate School of Medicine, 650-0017 Kobe, Hyogo Japan; Department of Mental Health, Nagoya University Graduate School of Medicine, 466-8550 Nagoya, Aichi Japan; Department of Neuropsychiatry, Kinki University Faculty of Medicine, 589-8511 Osaka-Sayama, Osaka Japan; Department of Social Rehabilitation, Kobe University School of Medicine, 654-0142 Hyogo, Japan; Department of Molecular Genetics, Brain Research Institute, Niigata University, 951-8585 Niigata, Japan; Pathology and Brain Disease Research Center, Brain Research Institute, Niigata University, 951-8585 Niigata, Japan

**Keywords:** CNV, Caudate, Genome instability, Schizophrenia, Somatic mutation

## Abstract

**Background:**

Cytogenomic mutations and chromosomal abnormality are implicated in the neuropathology of several brain diseases. Cell ﻿heterogeneity of brain tissues makes their detection and validation difficult, however. In the present study, we analyzed gene dosage alterations in brain DNA of schizophrenia patients and compared those with the copy number variations (CNVs) identified in schizophrenia patients as well as with those in Asian lymphocyte DNA and attempted to obtain hints at the pathological contribution of cytogenomic instability to schizophrenia.

**Results:**

Brain DNA was extracted from postmortem striatum of schizophrenia patients and control subjects (n = 48 each) and subjected to the direct two color microarray analysis that limits technical data variations. Disease-associated biases of relative DNA doses were statistically analyzed with Bonferroni’s compensation on the premise of brain cell mosaicism. We found that the relative gene dosage of 85 regions significantly varied among a million of probe sites. In the candidate CNV regions, 26 regions had no overlaps with the common CNVs found in Asian populations and included the genes (i.e., ANTXRL, CHST9, DNM3, NDST3, SDK1, STRC, SKY) that are associated with schizophrenia and/or other psychiatric diseases. The majority of these candidate CNVs exhibited high statistical probabilities but their signal differences in gene dosage were less than 1.5-fold. For test evaluation, we rather selected the 10 candidate CNV regions that exhibited higher aberration scores or larger global effects and were thus confirmable by PCR. Quantitative PCR verified the loss of gene dosage at two loci (1p36.21 and 1p13.3) and confirmed the global variation of the copy number distributions at two loci (11p15.4 and 13q21.1), both indicating the utility of the present strategy. These test loci, however, exhibited the same somatic CNV patterns in the other brain region.

**Conclusions:**

The present study lists the candidate regions potentially representing cytogenomic CNVs in the brain of schizophrenia patients, although the significant but modest alterations in their brain genome doses largely remain to be characterized further.

**Electronic supplementary material:**

The online version of this article (doi:10.1186/s13039-015-0144-5) contains supplementary material, which is available to authorized users.

## Background

Copy number variation (CNV) is defined as a deletion or duplication/multiplication of a genomic fragment spanning more than 1 kb when compared to a reference genome [1–3]. Approximately 37,000 sites of common CNVs have been identified in the human genome and they occupy 12 % of the entire genome [4, 5]. The genome-wide association studies (GWAS) on schizophrenia analyzed DNA which was isolated from peripheral lymphocytes and have identified risk CNV sites, some of which are not present in the patients’ parents [6–9].

Somatic mosaicism of genome sequences and structures have recently drawn particular attention [10–12]. Nearly 30 % of developing brain cells in human are reported to harbor aberrant chromosomal compositions [13, 14]. In addition, there are significant genomic differences in somatic cells between monozygotic twins and among tissues [15–18]. Accordingly, aberrant cytogenomic variations in human brain are implicated in neurodegenerative and neurodevelopmental disorders such as Alzheimer’s disease, amyotrophic lateral sclerosis, and Huntington’s diseases [19–25]. It is an open question whether the brain-specific somatic mutation or CNV might also contribute to the etiology or neuropathology of schizophrenia [26–28].

To obtain hints at the above question, we prepared DNA from the brain tissue of 48 schizophrenia patients and 48 control subjects. Labeling brain DNA samples, we directly applied those to Agilent 1 M comparative genomic hybridization (CGH) arrays to measure relative gene doses without the use of reference genome. This direct comparison through the case–control pairing reduces technical data deviations and enhances the statistical power of detection [29, 30]. With the potential genomic mosaicism of heterogeneous brain cell mixtures, we expected that the target genome could be diluted with normal DNA from the off-target cells and thus assumed non-integer values of CNVs in this analysis [31]. Technical limitations of this approach are further discussed below.

## Results

The striatum contain neural stem cells that proliferate throughout human life and carries somatic mutation in its mitochondrial genome [32, 33]. Therefore, we hypothesized that the striatum may be a potential candidate region that would exhibit somatic mosaicism in brain genome structures. DNA was extracted from postmortem striatum of patients with chronic schizophrenia (n = 48) and age-matched controls who had no history of neuropsychiatric disorders (n = 48) (Additional file 1: Table S1). Although there were significant differences in postmortem intervals (PMIs) between groups, there was no detectable difference in DNA quality (data not shown). All other indices were indistinguishable between schizophrenia patients and control subjects. A DNA sample was randomly picked from each group, paired to a sample in the other group, and subjected to two-color competitive CGH analysis with 1 M SurePrint G3 Human CGH Microarrays.

We applied the ADM-2 algorithm to the CGH signals of individual microarray probes (nearly 1 million) and searched for the primary candidate CNV loci associated with schizophrenia. A flowchart of the present study design is shown in Additional file 1: Figure S1. We chose1381 chromosomal loci that exhibited large group differences in gain/loss calls (Selection 1). In each probe site located on the primary candidate loci, we plotted the distribution of log2 signal ratios from 48 sets of microarray analyses and tested the null hypothesis that the mean log2 signal ratios was zero, indicating that the two groups were indistinguishable (Selection 2). We calculated total probabilities and averaged log2 signal ratios for individual candidate loci and judged their statistical significance with Bonferroni’s correction. The number of the candidate loci maintaining the statistical significance through Selection 2 was reduced to 85 (Details in Additional file 1: Table S2).

**Fig. 1:**
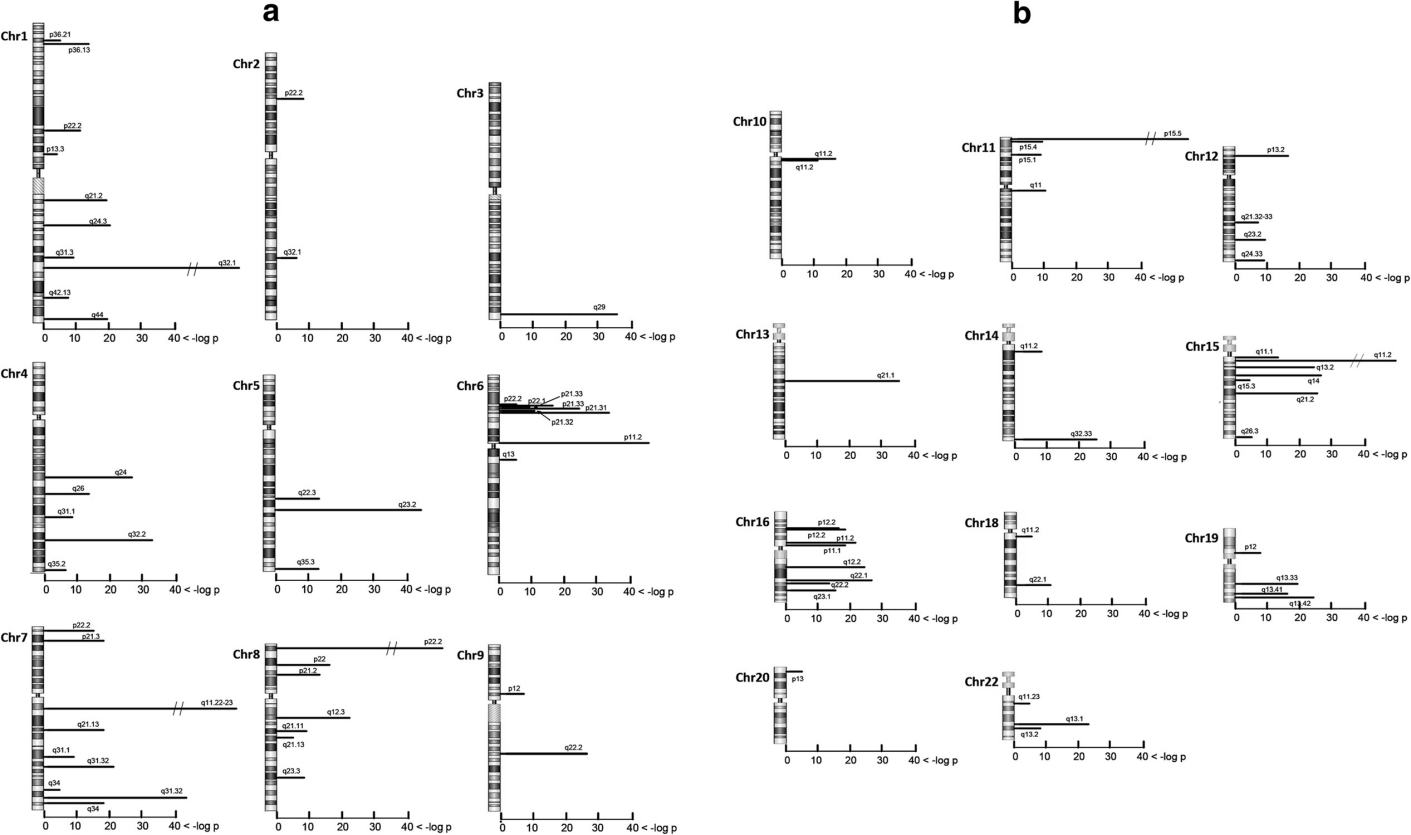
Statistically positive loci for log2 signal ratios in 48 sets of CGH microarrays. The signal bias was tested at each probe site by a two-tailed *t*-test. Bars represent the chromosomal position and lengths represent the logarithmic value of the probability of rejecting the null hypothesis. As comparisons were repeated 1381 times, we applied the Bonferroni’s correction and decreased a statistical threshold from *p* = 0.05 to *p* = 0.05/1381 (−log *p* = 4.44). Within a positive candidate CNV locus, neighboring probe sites were merged and their probabilities were cumulated (Additional file 1: Table S2). Note: Data from chromosomes 17 and 21 are not shown because these did not include any positives

Positive CNV loci were found in almost all chromosomes except chromosome 17 and 21 (Fig. 1). Individual loci covered 1–746 probe sites (3–2200 kb) and exhibited average log2 ratios of −1.46 to +0.63 (i.e., odds ratio (OR) = 0.36–1.55). A majority of the average log2 ratios were between −0.59 to +0.59 (i.e., < 1.5-fold differences) and only 4 loci showed more than 1.5-fold differences in array CGH signals. A genomic region spanning from 6p22.2 to 6p21.32 contained six CNV loci and included genes for the major histocompatibility complex that is highly associated with schizophrenia in GWAS [34]. Among the 85 CNV loci in Selection 2, 59 loci were reported and 26 loci were not reported in the CNV study on leukocyte DNA samples of Asian populations (Additional file 1: Table S2) [2].

To validate the authenticity of the present procedure, we attempted to verify the above genome dosage changes of several candidate loci using quantitative polymerase chain reaction (qPCR). According to the following two criteria, we selected the test loci whose signal differences were larger between groups and could be detectable with the given accuracy of qPCR; (i) those exhibiting the large and consistent gain/loss calls across the limited sample pairs (from Selection 1) and (ii) the loci represented larger global effects shared in most of the schizophrenia samples (from Selection 2).

**Fig. 2:**
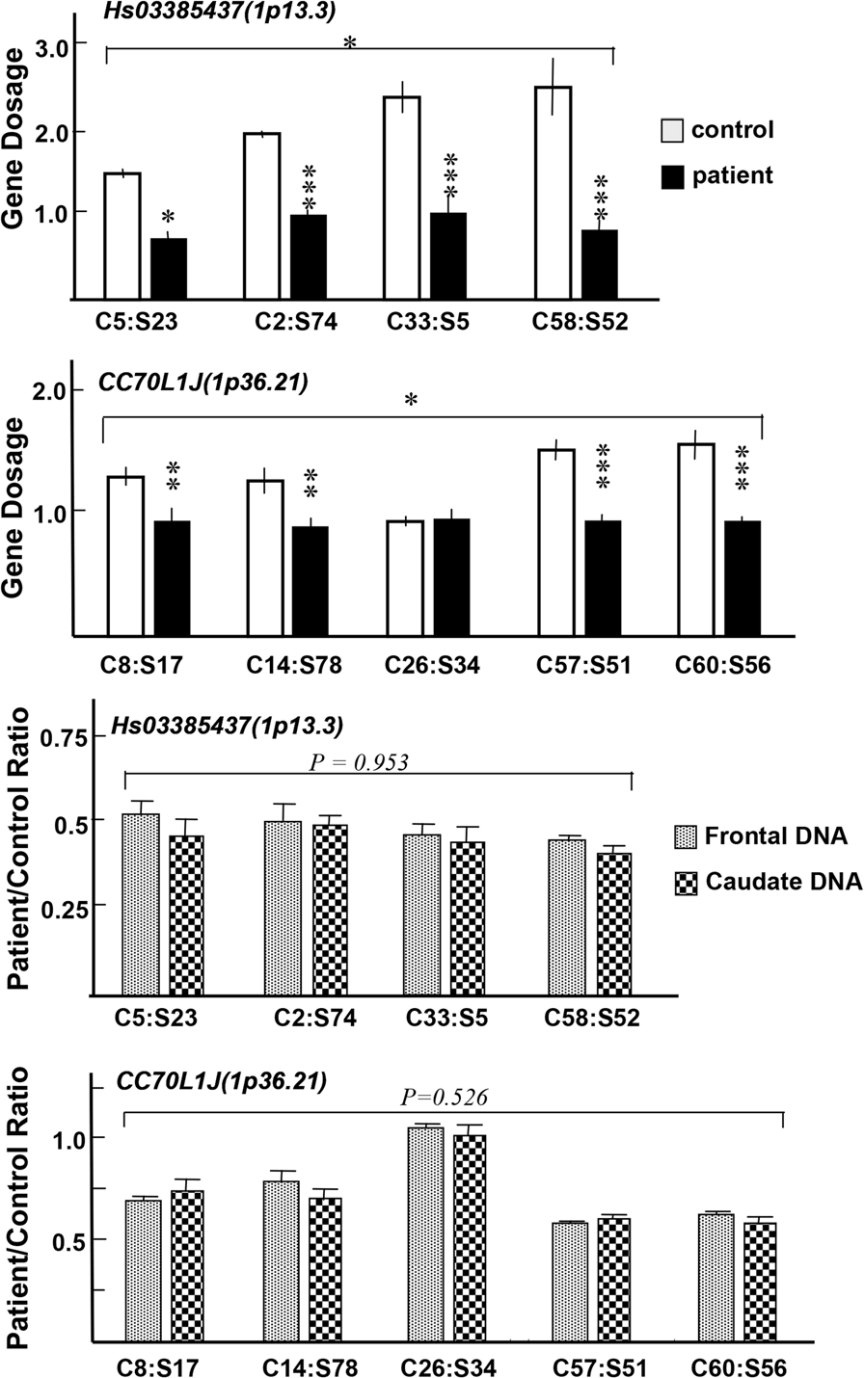
Gene dosage evaluation of the individual positive pairs in CGH analysis. DNA was prepared from the striatum and prefrontal cortex of the same subjects whose pairs resulted in the exclusive penetrance calls in array CGH analysis (Selection 1). DNA was then subjected to qPCR using the *RNase P* gene as an internal control in quadruplicate. Moreover, to compare the gene dosage between the striatum and prefrontal cortex, ratios of DNA dosages of the schizophrenia patients to those of the control subjects were calculated in each brain region and plotted. Statistical comparisons of gene dosages or their ratios were conducted by two-way ANOVA with the subject factors of disease and sample pair, considering technical deviations. *p < 0.05, **p < 0.01, ***p < 0.001

In the former category, the gene dosage of Hs03385437 (1p13.3), CC70L1J (1p36.21), Hs03318079 (Chr18:q22.1), Hs04794356 (4q24), Hs05080419 (9q22.2), and Hs07134106 (19p12) produced exclusive gain/loss calls in not less than four sample pairs. No discrepant calls were detected in any sample pairs. Using the same DNA pairs showing the difference in the penetrance call (Selection 1), we determined and confirmed the gene dosage of those DNA samples using qPCR. ANOVA detected significant gene dose differences at two loci (Hs03385437 and CC70L1J) between patient and control groups (Fig. 2).

In this measurement, we used *RNaseP* gene as an internal DNA dose control. Measured genome doses of the above regions appeared not to be integer levels in several control samples, potentially reflecting the cell mosaicism of the original tissues. We also extracted DNA from the prefrontal cortex of the same subjects of both groups and compared the genome doses of the above loci (Hs03385437 and CC70L1J). We calculated the copy number ratio of the patient’ DNA dosage to that of the control subject’ dosage and compared these ratios between the brain regions. At both loci, almost all the copy number ratios were markedly lower than 1.0 except the C26:S34 pair, supporting our primary observation that the absolute gene dosages of these loci were decreased in the schizophrenia samples. However, copy number ratios did not significantly differ between these brain regions in any of the sample pairs (Fig. 2). At least at these two candidate loci, we failed to find evidence for a gene dosage difference between these brain regions.

**Fig. 3:**
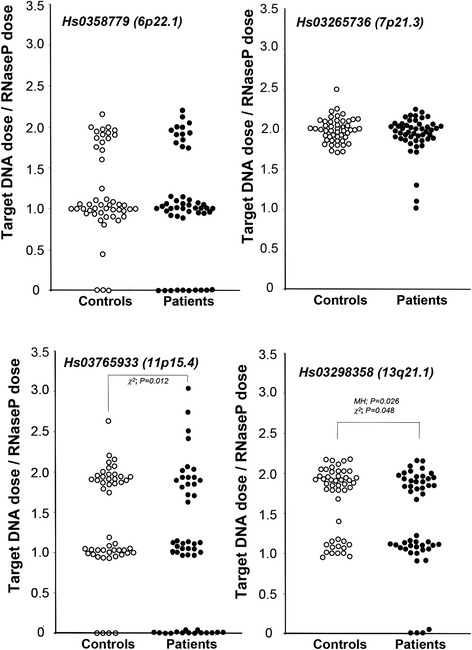
qPCR evaluations of candidate CNV loci from Selection 2. Gene dosages of the candidate loci 6p22.1, 7p21.3, 11p15.4, 13q21.1, and the *RNase P* (internal control) were determined by qPCR using all sample DNAs and TaqMan probes (Additional file 1: Table S3). Individual gene dosages of 48 patients’ or 48 controls’ DNAs were plotted and compared between groups using the Chi-square and Mann–Whitney U tests

In the latter category, Hs0358779 (6p22.1), Hs03265736 (7p21.3), Hs03765933 (11p15.4), and Hs03298358 (q21.1) exhibited higher log2 signal ratios and were thus subjected to the test evaluation. Gene dosage of these four loci were determined by qPCR using all the DNA samples in control and schizophrenia groups (n = 48 each). Differences in gene dosages were replicated by qPCR for Hs03765933 and Hs03298358 (Fig. 3). In contrast to the data distributions of Fig. 2, almost all the values of the gene doses were located at the levels of integers but with several exceptions. These candidate CNVs appear to reflect the gene dosage differences of germinal origin.

## Discussion

Several recent reports have indicated the neuropathological contribution of somatic CNV or DNA instability of the brain genome [19–28, 35–40]. In accordance with these findings, a small proportion few percent of brain cells are known to exhibit aneuploidy and carry large CNVs [13, 14, 41]. Aneuploidy is detected by fluorescence *in situ* hybridization (FISH) and appears to be increased by the onset of Alzheimer’s disease [20, 22]. The aneuploidy of chromosome 1, 18 and X was also identified in the brain of schizophrenia patients [21, 41]. Despite its advantages, FISH cannot be employed in exploratory investigations, unless the specific genome region of the CNV of interest is identified. Since bonafide genome structures from off-target cells could dilute the abnormal genome DNA population, more sensitive technologies remain to be developed, which detect low quantities of CNV in heterogeneous cell mixtures of the brain tissue [42, 43]. In the present study, we attempted to evaluate the efficacy of the CGH microarray technique to extract somatic CNVs in the postmortem brains of schizophrenia patients [42, 43].

With given semi-quantitative nature of the microarray technique, we applied statistics to the 1 M array CGH results from 48 sample pairs. Using the high density CGH array and statistical approach, we found 85 candidate CNV loci in the present study; 59 CNV loci are overlapped with the common CNV regions and the remaining 26 loci are not reported in peripheral leukocyte-derived DNA of Asian people [2, 44]. Of note, the 26 candidate regions encode the seven genes that are associated with or implicated in schizophrenia or other psychiatric diseases; ANTXRL, CHST9, DNM3, NDST3, SDK1, STRC, and SKY (Additional file 1: Table S2). DNM3 in the candidate region of 1q24.3 is disruptively mutated in some of schizophrenia patients [45]. ANTXRL and CHST9 are located in the CNV regions associated with bipolar disorder and autism [46, 47]. NDST3 and STRC are the risk genes for schizophrenia and hearing impairment that are identified by GWAS, respectively [48, 49]. SDK1 and SKY are the genes whose expression levels are markedly altered in the brain of schizophrenia patients [50, 51]. Accordingly, the present listing of the candidate brain CNVs is informative for future cytogenomic studies on schizophrenia [21, 41].

It was difficult for us to validate most of the above-mentioned 85 candidate loci with qPCR analysis with the given small signal differences between groups (i.e., less than 1.5-fold). Therefore, we selected the best 10 test loci that exhibited relatively large and/or wide effects on gene dosage. The six loci were chosen from Selection 1 as putative rare CNVs, which exhibited exclusive gain/loss calls in the limited number of samples. From Selection 2, the four loci were chosen as provisional common variants, which showed large effects and higher probability levels in the above parametric analysis. The qPCR analysis confirmed the schizophrenia-associated gene dosage differences at nearly half of the candidate CNV loci, suggesting the validity of the present strategy.

Unfortunately we had neither stored peripheral tissues nor information about these CNVs in peripheral DNA of the same subjects. To estimate the contribution of somatic CNVs to the present CNV listing, therefore, we were compelled to compare the gene dosages between the two brain regions or to search for their absence in the databases of Asian CNVs of leukocyte origin [2.44]. In the test PCR, however, we could not detect significant differences in gene dosages between the striatum and prefrontal cortex, at least, at these test CNV loci. If somatic CNVs were produced prior to neuroectodermal differentiation, there should be no difference between these two neural tissues, suggesting that the present comparison between these brain regions was inappropriate. Therefore, a comparative analysis of DNA from germinal cells of the same subjects will warrant this definitive conclusion [45].

Among the CNV candidate regions in Fig. 1, 26 candidate regions are not reported as the common CNVs of Asian populations [2, 44]. The majority of these loci exhibited high statistical significance with the probabilities of less than 10^−100^, such as 4q35.2, 6p11.2, 7q11–12, 11p15.4–15.5, and 15q11.2. In contrast, their CGH signal differences between patients and controls were markedly smaller (OR = 0.988–1.055). As discussed above, these candidate CNV loci include the peculiar genes that are implicated in schizophrenia [45–51]. These regions, which exhibited small signal differences, might represent more promising candidates of somatic CNV sites because the genome aberration of target cells is presumably diluted in the brain and should result in smaller ORs. However, such small differences in gene dosage should make the conventional qPCR verification more challenging with the given technical deviations [52]. To avoid target DNA dilution with cell mosaicism, single cell qPCR or FISH may be more beneficial in theory [20–22, 43]. However, it would be difficult to independently perform microdissection of hundreds of cells and perform single-cell analysis unless the target cell population is identified with molecular markers and its sensitivity of gene detection is high enough. FISH also requires properly fixed and processed brain tissues of the same subjects. With the given technical difficulties, therefore, we have been unable to verify these small variations.

## Conclusion

The present CGH analysis lists the potential candidate regions of somatic CNVs associated with schizophrenia, although most of those exhibited the modest but highly significant alterations in brain genome doses. Future studies aim to develop more elaborate techniques for somatic genome mosaicism and to verify the schizophrenia-associated cytogenomic instability in the above CNV candidates [53–56].

## Methods

### Ethical approval

The study was approved by Niigata University Medical Ethics Committee (No. 683). The use of postmortem brain tissues was authorized by the Matsuzawa Hospital Ethics Committee, Kobe University Medical Ethics Committee, Fukushima Medical University Ethics Committee, and Niigata University Medical Ethics Committee. The families of the control and schizophrenia patients provided written informed consent to allow the use of brain tissues for pathological investigations.

### Brain tissue

Postmortem brain tissue was collected from patients with chronic schizophrenia (30 men, 18 women; mean age, 64.5 ± 12.5 years old) and from age-matched control subjects (30 men, 18 women; mean age, 64.2 ± 12.0 years old), with no history of neuropsychiatric disorders (Additional file 1: Table S1). The diagnosis of schizophrenia was confirmed by examining the patient’s report according to DSM-III or DSM-IV categories (American Psychiatric Association). Postmortem brains of schizophrenia patients were collected at Matsuzawa Hospital, Kobe University, Fukushima Medical University and Niigata University, while those of control subjects were collected at Niigata University. In brief, the left cerebral hemisphere was fixed in formalin for diagnostic examination and the right hemisphere was frozen at −80 °C. Tissue samples were taken from postmortem brains that did not exhibit neurodegenerative abnormalities by conventional pathological staining (data not shown). The striatum (caudate) was identified in frozen coronal slices according to a human brain atlas. All tissues were collected and stored according to the principles of the Declaration of Helsinki, and tissue use was in compliance with the Human Tissue Act 2004.

### DNA extraction

High molecular weight DNA was extracted by the guanidinium − phenol procedure (Gentra Pure Gene Tissue Kit, Qiagen, Tokyo, Japan) according to the manufacturer’s protocol. Extracted DNA was quantified by spectrophotometry using a Nanodrop ND-2000® (Thermo Scientific Wilmington, DE, USA). Samples with absorbance ratios of A260/280 ~ 1.80 and A260/230  > 1.90, respectively, were regarded as sufficiently pure and suitable for CGH analysis. Some DNA samples were subjected to 1.0 % agarose gel electrophoresis for quality control. Evidence of DNA degradation was not detected in randomly-picked DNA samples from patient or control groups (data not shown).

### Comparative genomic hybridization (CGH)

Array-based CGH was performed by the manufacturer Takara Bio Dragon Genomics Center (Seta, Shiga, Japan). In brief, DNA (2 micro g) was fluorescent-labeled by random priming DNA synthesis in the presence of Cy3-dUTP (control group) or Cy5-dUTP (patient group) (Genomic DNA Enzymatic Labeling Kit; Agilent Technologies, Hachioji, Tokyo, Japan). DNA labeling efficiency was estimated by spectrophotometry (Nanodrop ND-2000®) measuring optical absorbance at 260 nm for DNA, at 550 nm for Cy5, and at 649 nm for Cy3. Cy5- and Cy3-labeled DNAs were randomly paired, mixed, and hybridized to SurePrint G3 Human CGH Microarrays (1 M) in the presence of human Cot-1 DNA (Oligo aCGH/ChIP-on-chip Hybridization Kit, Agilent Technologies). Following hybridization for 24 h, microarray slides were washed according to the manufacturer’s instructions and immediately scanned on a DNA Microarray Scanner (Agilent Technologies). With the given limitation of the sample number, we took an advantage of the above direct comparison between case and control samples [57]. This approach allowed us to determine relative ratios of their gene dosages but not their absolute gene dosages. However this procedure decreased data deviations, compared with the CGH analysis utilizing two microarrays and reference genome DNA [30].

### Quantitative polymerase chain reaction (qPCR)

To validate the results from the microarray experiments, we performed qPCR using TaqMan probes (Applied Biosystems, Foster City, CA) as described previously [32]. Gene dosages of the following genomic regions of interest were measured for the sample pair sets that exhibited the exclusive positive penetrance call with the Aberration Detection Method 2 (ADM-2) algorithm; CC70L1J (1p13.3), Hs03385437 (1p36.21), Hs04794356 (4q24), Hs05080419 (9q22.2), Hs03318079 (18q21.1), and Hs07134106 (19p12). Using all the samples, we also determined the gene dosages of the candidate CNV loci that exhibited lower probability scores by the global *t*-test analysis; Hs03587795 (6p22.1), Hs03265736 (7p21.3), Hs03765933 (11p15.4), and Hs03298358 (13q21.1). DNA sequences of TaqMan probes and PCR primers are shown in the Additional file 1. We obtained cycle threshold (CT) values for the region of interest for each sample with FAM™-labeled probes, simultaneously monitoring those for *RNaseP* gene (an internal control) with its VIC®-labeled probe (ABI PRISM 7900HT Sequence Detection System and SDS v2.3 software, both Applied Biosystems). These CT values of the target gene and *RNaseP* gene were obtained for all the DNA samples. Copy number of the target gene was estimated from CT values by CopyCaller v1.0 software (Applied Biosystems).

### Statistics

The ADM-2 algorithm prompted by Genomic Workbench software (edition 5.0.14, Agilent Technologies, 2010) was used to identify individual and common aberrations for 48 microarray data sets. This algorithm identifies all aberrant intervals with consistently high or low log ratios based on the statistical score. The algorithm searches for intervals where a statistical score based on the average quality-weighted log ratio of the sample and reference channels exceed a user-specified threshold. For the primary screening (Selection 1), we applied the following filtering options to the human genome assembly hg19 (excluding sex chromosomes): sensitivity threshold = 6, fuzzy zero = On, bin size = 10, and centralization threshold = 6. We then selected the primary candidate loci of somatic CNVs which exhibited > =4 difference in gain/loss calls in the whole penetrance summary.

To calculate mean signal OR and the probability of CNVs between groups, we plotted individual log2 signal ratios at all the probe sites within the above candidate loci. The Kolmogorov − Smirnov test revealed that log2 signal ratios were judged to fit into the Gaussian distribution at more than 80 % of probe sites. Assuming their Gaussian distribution, we analyzed their statistical biases against log2 = 0 (i.e., the null hypothesis of equal signal intensities between patients and controls) by two tailed *t*-test at each probe position. Within a candidate CNV locus containing multiple probe sites, their log2 signal ratios were averaged and probabilities were summed and then subjected to Bonferroni’s correction (Selection 2). Statistical difference of qPCR results between individual sample pairs was determined with ANOVA or two tailed *t*-test, incorporating technical errors into account. Alternatively, group differences of qPCR results from individual samples were estimated by the chi-square and Mann–Whitney U tests. Statistical analyses were performed using SPSS software (IBM Japan, Tokyo, Japan).

## Additional file

Additional file 1: Figure S1. A flowchart of the study design. **Table S1.** Autopsy and clinical information of the subjects used. **Table S2.** List of candidate CNV regions and their statistical details. **Table S3.** Custom Taqman PCR primers and probes used.

## Abbreviations

ADM: Aberration detection method
ANOVA: Analysis of variance
CGH: Comparative genomic hybridization
CNV: Copy number variation
CT: Cycle threshold
FISH: Fluorescent in situ hybridization
OR: Odds ratio
PMI: Postmortem interval
qPCR: Quantitative polymerase chain reaction
