# Supplementary material for: Assessment of copy number variations in the brain genome of schizophrenia patients
Source: Mol Cytogenet. 2015 Jul 1;8:46. doi: 10.1186/s13039-015-0144-5 (PMC4487564; doi:10.1186/s13039-015-0144-5)
Supplement: Additional file 1: Figure S1. — A flowchart of the study design. Table S1. Autopsy and clinical information of the subjects used. Table S2. List of candidate CNV regions and their statistical details. Table S3. Custom Taqman PCR primers and probes used. [file 13039_2015_144_MOESM1_ESM.pdf]

Supplemental information

M Sakai et al;

Assessment of Copy Number Variations in The Brain Genome of Schizophrenia Patients

Figure S1.

A flowchart of the study design

Table S1.

Autopsy and clinical information of the subjects used

Table S2.

Statistical details of array CGH signals

Table S3.

Custom Taqman PCR primers and probes designed by the authors.

Note; Illumina Co Ltd does not disclose the sequences of the other primers and probes

Figure S1. A flowchart of the study design

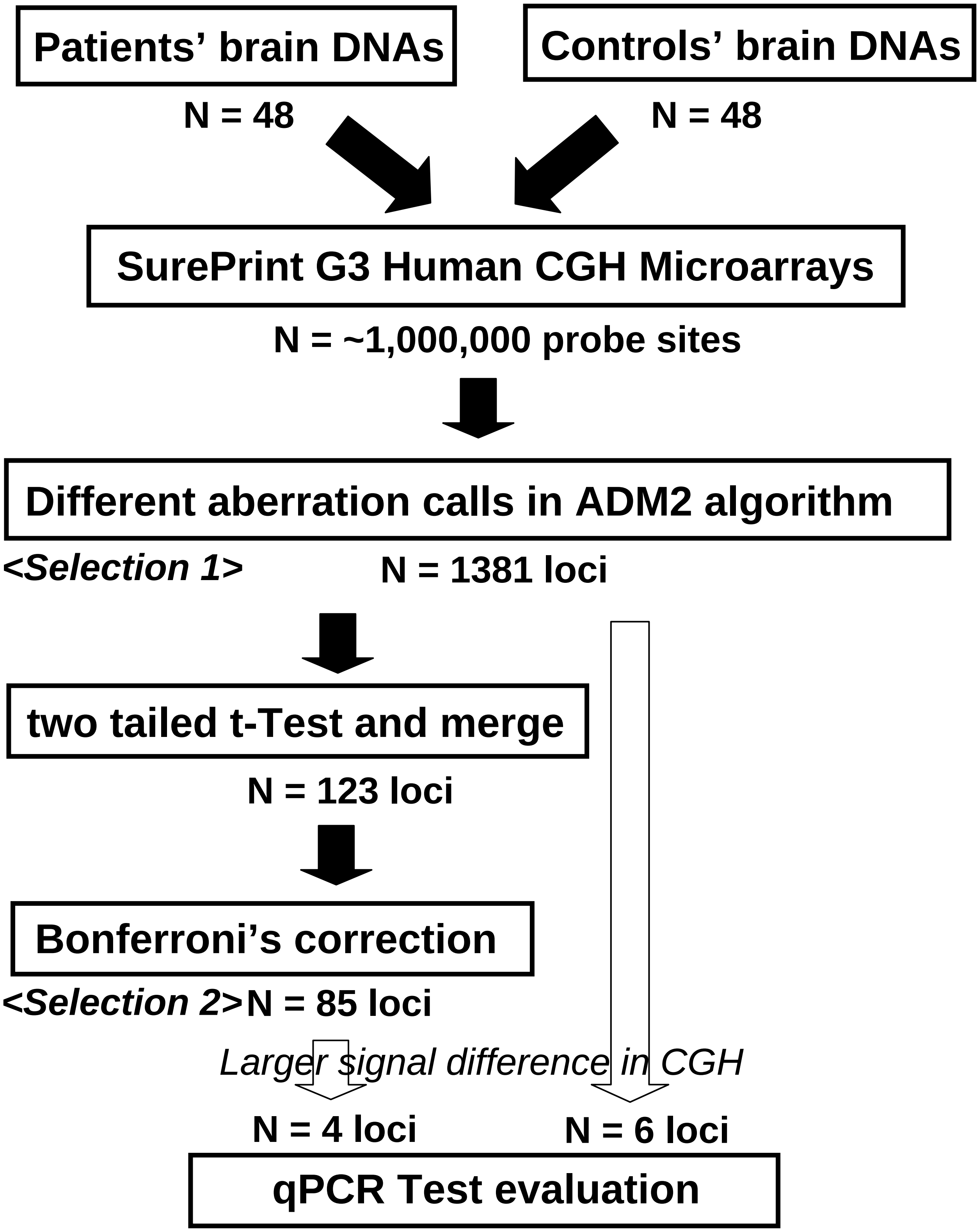

Autopsy and clinical information of the subjects used

schizo	gender	age	cause of death	PMI	brain weight
1	M	68	Bleeding	2	UN
2	M	59	Myotonic dystrophy	12	UN
3	F	63	unidentified	2	UN
4	M	72	unidentified	4	UN
5	M	49	gastric cancer	11	UN
6	F	64	decbihypoglicemia	21	1380
7	M	50	renal failure	2	1155
8	F	56	acute heart failure	2	1220
9	M	49	gastric cancer	UN	UN
10	M	59	Myotonic dystrophy	12	1120
11	M	72	unidentified	UN	1310
12	M	51	unidentified	5	1210
13	F	72	liver failure	UN	UN
14	F	62	unidentified	4	1220
15	M	36	unidentified	16	1740
16	M	88	unidentified	UN	1340
17	M	72	ileus	UN	UN
18	F	83	unidentified	3	930
19	F	85	unidentified	UN	UN
20	M	51	renal failure	UN	1500
21	F	72	unidentified	10	1405
22	F	83	unidentified	13	1135
23	M	48	gastric cancer	UN	UN
24	M	70	Bile duct cancer	31	1334
25	M	75	pneumonia	19	1157
26	M	64	pneumonia	6.5	1310
27	F	68	Chronic renal failure	16	1297
28	M	60	acute myocardinal infarction	46	1316
29	F	77	pneumonia	26	1301
30	M	74	pneumonia/heart failure	20.5	1114
31	M	71	pneumonia	17.5	1185
32	M	39	suicide	35	1260
33	M	58	stomach cancer	10	1320
34	M	56	pancreatic cancer	1.5	1280
35	F	53	Cardiac Failure	5	1090
36	F	66	ascending cancer	3.5	1110
37	M	77	Chronic renal failure	5	1305
38	F	76	Pneumonia	8.5	1100
39	F	59	Pneumonia	2.5	1350
40	M	57	pancreatic cancer	45.5	1340
41	M	78	gall stone , pneumonia	13	1355
42	F	70	Pick disease	16.5	1210
43	M	59	unidentified	18.43	1290
44	M	76	unidentified	26.5	1280
45	M	63	colonal cancer	UN	1300
46	M	65	gastric cancer	20	UN
47	F	38	ovarian tumor	UN	UN
48	F	83	unidentified	3	930
	M 30	Mean 64.2	Mean	13.6	1256
	F 18	SD 12.0	SD	11.7	148
			Note; UN represents “unknown”		

Control	gender	age	cause of death	PMI	Brain Weight
1	F	35	Chronic myeloblastic leukemia	2	1255
2	M	67	Hemophagocytosis	1	1220
3	F	49	Sudden death	1	1075
4	M	82	Mixed connective tissue disease	2	1150
5	F	61	Pneumonia	3	1335
6	M	77	Myelodysplastic syndrome	22	1175
7	F	75	Cushing's disease	15	1140
8	F	55	Adult T-cell leukemia	4	1230
9	M	64	Renal failure	3.5	1240
10	M	51	Acute abdomen	5.5	1320
11	M	41	Ducchene muscular dystrophy	4	1380
12	M	48	Myotonic dystrophy	7	1450
13	F	53	Myotonic dystrophy	4	1100
14	M	76	Herpes encephalitis, chronic stage	1.5	1230
15	F	73	Myositis	4	1010
16	M	74	Esophageal cancer	2	1270
17	M	56	Marinesco-Sjagren syndrome	20	1180
18	M	68	Myopathy	4	1220
19	M	71	Multiple small infarcts	3	1280
20	F	44	Gorham's disease	6	1240
21	M	51	pontine hemorrhage	5	1550
22	F	64	polymyositis	2	1150
23	F	48	Myastenia gravis	2	1150
24	M	75	pathymeningitis	2	1105
25	F	70	Multiple cerebral infarcts	2	1105
26	M	68	Cbr.infarction	3	1205
27	M	84	Decrepitude	13	UN
28	M	70	gastric cancer	5	UN
29	M	77	gastric cancer	6	UN
30	M	65	pulmonary infarction	6	UN
31	F	49	Crow-Fukase syndrome	2	1135
32	M	55	myotonic distrophy	4.5	1150
33	F	86	SCA6	2	990
34	F	64	polymiositis	2	1150
35	F	79	Foix-Alajournine	2.5	1100
36	M	71	infarcts	UN	1365
37	M	59	Myeloma	2	1280
38	F	60	Myopathy	3	1145
39	M	76	GI bleeding	3.5	1270
40	F	49	Myopathy	3	1070
41	F	42	Fresh muscle necrosis, tetanus	6.5	1115
42	M	70	Multiple fresh infarcts	3.5	1070
43	M	71	Crow-Fukase symdrome	3.5	1065
44	M	72	Myasthenia gravis	2.5	1420
45	M	68	Lung cancer	3	1205
46	M	60	small infarcts	2	1390
47	M	82	Multiple infarcts, gastric cancer	6	1195
48	M	75	pathymeningitis	2	1105
	M 30	Mean 64.2	Mean	4.5	1204
	F 18	SD 12.6	SD	4.4	120

Supple Table S1

Statistical details of array CGH signals

Column Definitions
1) locus; chromosomal region with positive aberrations in aCGH
2) start/stop; nucleotide region showing the positive aberrations in hg 19
3) r; reported, n; not reported; the CNV region is reported or not reported in the Database of Genomic Variant
4) prove; the number of probes showing positive aberrations in the above region
5) (-logp) AVE; the mean of logarithmic probabilities of individual aberrations
6) (-logp) AVE; the standard deviation of logarithmic probabilities of individual aberrations
7) total -logp; cumulative logarithmic probability in which the mean of individual signal ratios is indistinguishable from 1.000
8) log2ratioAVR; averaged logarithmic signal odd ratios for the chromosomal region

	locus	start	stop	reported/not reported	probe	total -logp	(-logp) AVR	(-logp) SD	Log_2 ratio AVR	
Chr1										
1	p36.21	13178469	13178528	r	1	5.02	—	—	-0.34	
2	p36.13	17207777	17241809	r	5	13.10	2.66	1.47	-0.21	
3	p22.2	89263381	89302718	r	11	11.24	3.36	1.44	-0.04	
4	p13.3	109578043	109578102	r	1	4.87	—	—	-0.23	
5	q21.2	149144534	149205091	r	7	18.79	2.68	1.92	0.17	
6	q24.3	172114109	172115477	n	3	20.04	6.68	3.91	0.21	DNM3
7	q31.3	196747506	196796229	r	4	9.22	2.31	0.40	-0.20	
8	q32.1	202346831	202534440	r	19	76.81	4.43	2.27	0.13	
9	q42.13	229812529	229816231	r	4	7.67	1.92	0.32	0.63	
10	q44	248624618	248745861	r	8	19.35	2.42	1.13	-0.20	
Chr2										
1	p22.1	41239485	41240851	r	2	8.05	4.02	3.28	-0.26	
2	q32.1	185754844	185772343	r	3	6.83	4.02	1.90	-0.15	
Chr3										
1	q29	195419168	195478558	r	10	35.76	3.58	1.47	0.25	
Chr4										
1	q24	107065443	107065502	n	1	26.63	—	—	0.33	
2	q26	114345582	119127415	n	4	13.26	3.32	1.35	-0.23	NDST3
3	q31.1	140949472	140955970	n	2	7.68	3.84	3.26	-0.03	
4	q32.2	161861549	161931883	r	10	32.23	3.22	1.30	-0.26	
5	q35.2	190471322	190659488	r	23	6.28	4.48	3.25	-0.004	
Chr5										
1	q22.3	114711767	114721036	n	3	12.63	4.21	2.12	0.18	
2	q23.2	126595033	126595033	n	6	44.09	9.33	2.73	0.25	
3	q35.3	180410254	180424663	r	4	12.65	3.16	3.43	0.32	
Chr6										
1	p22.2	26745191	26753264	r	2	4.66	2.33	0.93	-0.28	
2	p22.1	29854870	29896710	r	7	16.07	2.30	1.43	-0.46	
3	P21.33	31117019	31188599	n	2	8.94	4.47	0.43	0.18	
4	p21.33	31281393	31451139	r	17	24.58	3.96	3.57	-0.09	
5	p21.32	32525713	32634848	r	9	11.01	2.08	0.62	0.26	
6	p21.31	35323974	35767193	r	9	33.12	3.68	2.05	0.24	
7	p11.2	57436765	57581950	n	31	44.95	6.54	6.69	0.03	
8	q13	74709760	74711681	r	2	4.97	2.48	0.70	0.21	

Supple Table S2-2

	locus	start	stop	reported/not reported	probe	total -logp	(-logp) AVR	(-logp) SD	Log_2 ratio AVR	
Chr7										
1	p22.2	3440049	3441459	n	2	15.48	7.74	8.00	-0.16	
2	p21.3	11313242	11342156	n	8	16.51	2.91	2.53	-0.06	SDK1
3	q11.22<br>q11.23	72026466	72315358	r	29	77.81	4.01	2.09	-0.08	
4	q21.13	90186224	90220600	r	11	18.30	4.06	1.84	0.05	
5	q31.1	111035795	111037250	r	2	8.73	4.37	4.11	-0.21	
6	q31.32	121165229	121175668	r	4	21.07	5.27	2.52	-0.20	
7	q34	141766883	141786368	r	2	4.71	2.36	1.06	-0.74	
8	q36.1	149779296	149859892	n	9	43.09	5.09	4.54	-0.16	
9	q36.2	153672608	153742314	r	6	17.26	3.81	1.11		
Chr8										
1	p23.2	3734470	3816456	r	26	79.40	3.23	2.01	-0.14	
2	p22	15403439	15413466	r	3	15.73	5.24	0.58	-0.31	
3	p21.2	25411694	25414740	r	3	13.14	4.38	2.75	-0.29	
4	q12.3	63352266	63397172	n	11	21.40	2.49	1.02	0.10	
5	q21.11	77161734	77188957	n	2	9.68	4.84	1.07	0.30	
6	q21.13	83918000	83929768	n	2	5.99	3.00	2.52	-0.15	
7	q23.3	112323159	112340290	n	3	8.18	2.73	1.88	0.23	
Chr9										
1	p12	43590080	43686974	r	3	6.40	2.13	1.27	0.25	
2	q22.2	93564261	93564318	n	4	27.64	6.48	0.33	-0.23	SKY
Chr10										
1	q11.2	46969728	46971695	r	2	16.03	8.01	3.16	0.20	
2	q11.2	47610149	47697197	n	12	10.58	3.55	1.36	0.02	ANTXRL
Chr11										
1	p15.5<br>p15.4	262349	3167131	r	746	3470.71	6.59	5.93	0.08	
2	p15.4	4970007	4975697	r	5	9.49	1.90	0.36	-0.98	
3	p15.1	18949929	18960666	r	4	9.02	2.25	0.55	0.22	
4	q11	55433570	55444227	r	2	10.42	5.21	1.95	-0.12	
Chr12										
1	p13.2	10574520	11249210	r	6	15.76	3.15	2.30	-0.18	
2	q21.31<br>q21.32	86695679	86703030	r	3	7.19	2.40	0.79	-0.16	
3	q23.2	102477092	102477151	n	4	8.86	2.22	0.50	-0.18	
4	q24.33	131797898	131802404	r	2	8.87	4.44	4.10	0.16	
Chr13										
1	q21.1	57759370	57783723	r	13	34.53	2.66	0.77	-0.62	
Chr14										
1	q11.2	24267085	24271075	r	3	7.70	2.57	0.94	-0.14	
2	q32.33	106602112	106727130	r	5	24.90	4.98	4.35	-0.37	

Supple Table S2-3

	locus	start	stop	reported/not reported	probe	total -logp	(-logp) AVR	(-logp) SD	Log_2 ratio AVR	
Chr15										
1	q11.1	20549990	20616722	r	5	13.02	2.60	1.57	0.27	
2	q11.2	20863313	22551703	r	61	122.55	2.54	2.01	0.25	
3	q13.2	30652489	31043038	r	14	22.63	2.87	1.30	-1.46	
4	q14	34730547	34794497	r	10	25.88	2.59	1.06	-0.32	
5	q15.3	43895633	43895690	n	1	4.84	—	—	0.34	
6	q21.2	49652586	49684919	n	10	25.11	3.79	2.18	-0.05	
7	q26.3	102480829	102480888	r	1	4.79	—	—	-0.20	
Chr16										
1	p12.2	21475039	21593631	r	4	15.96	3.99	3.68	-0.18	
2	p12.2	22645706	22697409	r	5	17.59	3.52	1.89	-0.21	
3	p11.2	32471625	33923140	r	10	21.26	2.13	0.46	0.07	
4	p11.1	35024449	35055525	n	6	18.51	3.08	1.36	0.06	
5	q12.2	55806212	55811275	r	3	24.34	8.11	3.31	0.23	
6	q22.1	70052979	70280942	r	7	26.90	3.84	2.25	-0.13	
7	q22.2	72083919	72097048	r	3	13.15	4.38	1.72	0.19	
8	q23.1	77206239	77224028	r	4	15.35	3.84	2.07	-0.15	
Chr17										
none										
Chr18										
1	q11.2	24756700	24758882	n	2	4.45	2.23	1.01	0.13	CHST9
2	q22.1	66742452	66742511	n	1	10.50	—	—	-0.19	
Chr19										
1	p12	20718947	20719006	n	1	7.48	—	—	-0.19	
2	q13.33	49183320	49206305	n	4	19.14	6.20	8.17	-0.19	
3	q13.41	53328041	53352736	r	5	16.04	3.71	1.54	-0.15	
4	q13.42	54732917	54742404	n	3	23.68	9.59	6.80	0.17	
Chr20										
1	p13	1573410	1583221	r	1	4.77	1.59	0.06	-0.77	
Chr21										
none										
Chr22										
1	q11.23	25664618	25667114	r	2	4.63	2.31	1.23	0.18	
2	q13.1	39291820	39385485	r	11	21.87	2.31	1.24	0.33	
3	q13.2	42896699	42907693	r	2	7.74	7.29	5.47	0.10	

Supple Table S3

Custom Taqman PCR primers and probes used

Oligo DNA sequences in Figure 2

chromosome		start	stop	Assay number	RT-PCR assay	DNA sequence
chr6	p22.1	29854870	29896710	Hs03587795_cn	recepter	CAGGAGAATGTTCCTGCTGAGGACA
					F primer	AppliedBiosystems designed but unpublished
					R primer	AppliedBiosystems designed but unpublished
chr7	p21.3	11313242	11342156	Hs03265736_cn	recepter	GATGAGAAATTTTGCCGCTGTTTGA
					F primer	AppliedBiosystems designed but unpublished
					R primer	AppliedBiosystems designed but unpublished
chr11	p15.4	4970007	4975697	Hs03765933_cn	recepter	TACTGCCTATTTGTCTGCCCACGAT
					F primer	AppliedBiosystems designed but unpublished
					R primer	AppliedBiosystems designed but unpublished
chr13	q21.1	57759370	57783723	Hs03298358_cn	recepter	GGCAGACAAGTATGGGTGCTAAATC
					F primer	AppliedBiosystems designed but unpublished
					R primer	AppliedBiosystems designed but unpublished

Oligo DNA sequences in Figure 3

chromosome		start	stop	Assay number	RT-PCR assay	DNA sequence
chr1	p13.3	109578043	109578102	Hs03385437_cn	recepter	GGTAAGCCTTCCTACTCCAGTCAGG
					F primer	AppliedBiosystems designed but unpublished
					R primer	AppliedBiosystems designed but unpublished
chr1	p36.21	13178469	13178528	CC70L1J	recepter	CCTAGATAGTGTTCCAGACTCCC
					F primer	CCATCTACATAATGCATGTCAAACCAA
					R primer	GCCTAGAAAACATTAGTGTAACTGGGT
chr4	q24	107065443	107065502	Hs04794356_cn	recepter	TGTCCCAAATTTGGAACTGATATAC
					F primer	AppliedBiosystems designed but unpublished
					R primer	AppliedBiosystems designed but unpublished
chr9	q22.2	93564261	93564318	Hs05080419_cn	recepter	AAAATACAGGTGGGTTCCGCCAGCT
					F primer	AppliedBiosystems designed but unpublished
					R primer	AppliedBiosystems designed but unpublished
chr18	q22.1	66742452	66742511	Hs03318079_cn	recepter	GAACTGGGAATACGACTCCTTGAGG
					F primer	AppliedBiosystems designed but unpublished
					R primer	AppliedBiosystems designed but unpublished
chr19	p12	20718947	20719006	Hs07134106_cn	recepter	TTTTATCCGCTTCACTTCCATTCAC
					F primer	AppliedBiosystems designed but unpublished
					R primer	AppliedBiosystems designed but unpublished
